# Outbreak of Henipavirus Infection, Philippines, 2014

**DOI:** 10.3201/eid2102.141433

**Published:** 2015-02

**Authors:** Paola Katrina G. Ching, Vikki Carr de los Reyes, Maria Nemia Sucaldito, Enrique Tayag, Alah Baby Columna-Vingno, Fedelino F. Malbas, Gilbert C. Bolo, James J. Sejvar, Debbie Eagles, Geoffrey Playford, Erica Dueger, Yoshihiro Kaku, Shigeru Morikawa, Makoto Kuroda, Glenn A. Marsh, Sam McCullough, A. Ruth Foxwell

**Affiliations:** Department of Health, Manila, Philippines (P.K.G. Ching, V.C. de los Reyes, M.N. Sucaldito, E. Tayag, F.F. Malbas Jr.);; Center for Health Development Region XII, General Santos, Philippines (A.B. Columna-Vingno);; Department of Agriculture, Manila (G.C. Bolo, Jr);; World Health Organization, Manila (J.J. Sejvar, D. Eagles, G. Playford, E. Dueger, A.R. Foxwell);; Centers for Disease Control and Prevention, Atlanta, Georgia, USA (J.J. Sejvar, E. Dueger);; Australian Animal Health Laboratory, Geelong, Victoria, Australia (D. Eagles, G.A. Marsh, S. McCullough);; University of Queensland, Brisbane, Queensland, Australia (G. Playford);; National Institute of Infectious Disease, Tokyo, Japan (Y. Kaku, S. Morikawa, M. Kuroda);; Australian National University, Canberra, Australian Capital Territory, Australia (A.R. Foxwell)

**Keywords:** outbreak, henipavirus, emerging disease, viruses, Philippines

## Abstract

During 2014, henipavirus infection caused severe illness among humans and horses in southern Philippines; fatality rates among humans were high. Horse-to-human and human-to-human transmission occurred. The most likely source of horse infection was fruit bats. Ongoing surveillance is needed for rapid diagnosis, risk factor investigation, control measure implementation, and further virus characterization.

Henipaviruses belong to a genus of recently emerging viruses within the family *Paramyxoviridae* (*1*–*3*) and include 2 zoonotic members: Hendra virus (HeV) and Nipah virus (NiV). HeV was first described in Australia in 1994, when it caused an outbreak of severe acute respiratory diseases that led to a high mortality rate among horses. Subsequently, several sporadic cases of HeV infection have occurred in horses in Australia; transmission to humans has occurred and the fatality rate was high (*4*,*5*). NiV was first recognized as a human pathogen in peninsular Malaysia in 1998. This outbreak among pig farmers and abattoir workers exposed to infected swine secretions (*6*) was associated with severe encephalitic illness and a high fatality rate. Subsequently, NiV emerged as a major public health problem in Bangladesh and India (*7*–*9*).

The natural reservoir of both viruses is pteropid bats, which harbor the viruses but do not show clinical illness (*3*). Virus transmission from bats to domestic animals is thought to be through pasture or feed contaminated by bat urine, feces, or other excretions (*10*). Transmission of HeV to humans has been invariably associated with close contact with ill horses (*4*), and transmission of NiV in Bangladesh is mainly through date palm sap contaminated with bat secretions (*11*). Human-to-human transmission of NiV also occurs (*12*,*13*).

## The Study

On April 2, 2014, the Philippine National Epidemiology Center received a report of human deaths in 2 villages, Tinalon and Midtungok, in the municipality of Senator Ninoy Aquino, province of Sultan Kudarat, island of Mindanao. The villages are ≈15 km apart, and the provincial referral hospital is in Isulan, 80 km away. An outbreak investigation led by the National Epidemiology Center identified additional human deaths and nonfatal infections and concurrent neurologic disease and sudden deaths in several horses, all of which were subsequently consumed by villagers. On May 12, 2014, the Philippine government asked the World Health Organization for further outbreak investigation assistance.

During May 22–24, 2014, a combined team from the Philippine Department of Health, Department of Agriculture, and the World Health Organization interviewed persons who survived, those with suspected cases, and family members of the deceased and conducted focus group interviews with other persons in affected villages. Key informants from local human and animal health agencies were also interviewed, and hospital records for persons with suspected cases were reviewed. We defined a human case as illness in any person with an epidemiologic link to the municipality of Senator Ninoy Aquino and who had experienced acute encephalitis syndrome, severe influenza-like illness (ILI), or meningitis during March 3–May 24, 2014.

**Figure 1:**
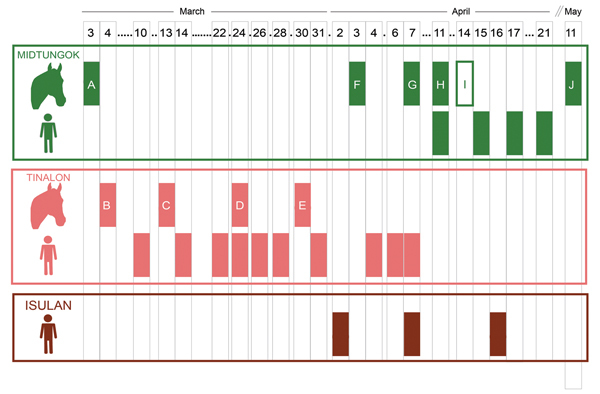
Temporal and geographic features of human and horse cases in 2 villages (Midtungok, Tinalon) and at the provincial referral hospital (Isulan), Philippines, 2014, by date of disease onset. Full rectangles represent cases based on case description. The empty rectangle (horse I) represents the horse death that did not fit the case description.

The case definition was met by 17 persons (11 acute encephalitis syndrome, 5 ILI, 1 meningitis). Clinical signs developed for the index case-patient on March 10 and for the last case-patient on April 21 (Figure 1). The case-fatality rate among those with acute encephalitis syndrome was 82%; no patient with ILI or meningitis died. Of acute encephalitis syndrome survivors, 1 experienced residual severe cognitive impairment, motor weakness, and ataxia, and the other experienced persistent ophthalmoplegia. Median incubation period for case-patients with known exposure was 8 days.

Of the 17 case-patients, a total of 7 (41%) had participated in horse slaughtering and horse meat consumption, and 3 (18%) had only consumed horse meat and had no history of slaughtering or meat preparation (Table 1). Five (29%) case-patients had been exposed to other human case-patients but not to any horses. Of these, 2 were health care workers from Isulan who did not visit the villages, had no contact with sick horses, and did not consume horse meat (Figure 1); they reportedly wore minimal personal protective equipment (gloves, face mask) during patient procedures, 2 cared for case-patients in their homes, and 1 helped transport a case-patient (who was producing substantial respiratory secretions) to a hospital.

**Table 1:** Exposure and infection profile of henipavirus case-patients, Sultan Kudurat, Mindanao, Philippines, March 3–May 24, 2014

Clinical presentation	Slaughter and meat consumption	Meat consumption alone	Exposure to probably infected human	Uncertain exposure	Total
Acute encephalitis syndrome					
No. patients	3	3	4	1	11
Sex, M:F	3:0	3:0	4:0	1:0	11:0
Age , y	21, 32, 60	30, 51, 54	24, 29, 35, and 46	28	32 (median)
Incubation period	6, 8, 8	7, 10, 20	3–8. 6, 7, 8	Unknown	3–20
No. deaths	3	2	3	1	9
Influenza-like illness (n = 5) or meningitis (n = 1)					
No. patients	4	0	1	1	6
Sex, M:F	4:0	NA	1:0	0:1	5:1
Age, y	21, 23, 26, 39	NA	46	26	26 (median)
Incubation period, d	7, 9, 15, 15	NA	4	Unknown	4–15
No. deaths	0	NA	0	0	0

*NA, not applicable.

During March 3–May 11, ten horse deaths were reported in the 2 villages (Figure 1); 2 were found dead, and all but 1 of the others showed neurologic signs (head tilting, circling, ataxia). Progression of clinical signs was rapid. Among other domestic animals, 4 cats that had eaten horse meat died within 5 days of their probable exposure date; 3 were found dead and the other exhibited terminal bleeding from the nose and/or mouth. A dog was found dead after eating horse meat, but the epidemiologic link is unknown.

Blood was collected from surviving suspected case-patients, contacts of human or horse case-patients, and several domestic animals (cats, buffalo, dogs, horses, pigs, goats). Retrospectively collected cerebrospinal fluid (n = 2) and serum (n = 7) samples from persons with suspected cases underwent further testing. No samples were available from affected horses.

Testing for a range of neurotropic pathogens was conducted at the Australian Animal Health Laboratory and the National Institute of Infectious Diseases (Japan). Test results were negative for all agents except henipaviruses.

**Figure 2:**
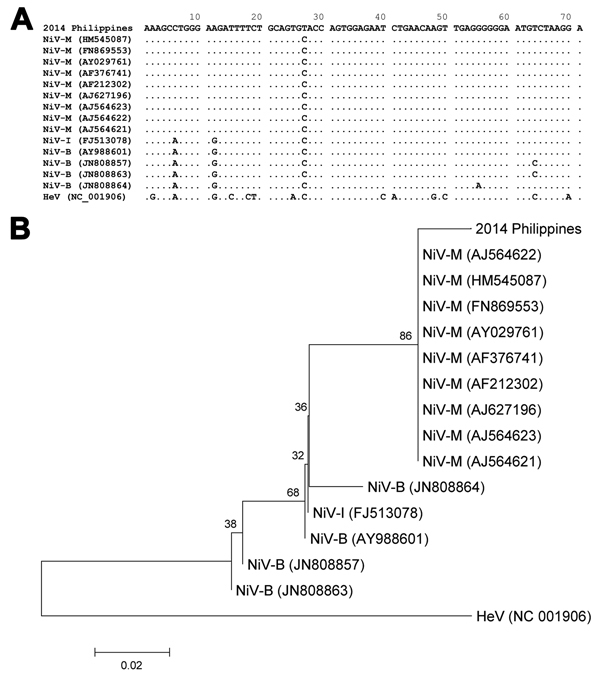
Alignment (A) and phylogenetic relationship (B) of partial phosphoprotein gene sequences (71 mer) of henipaviruses, including the fragment obtained by next-generation sequencing from a patient in Philippines (2014 Philippines). The alignment was conducted by using the MUSCLE program (http://www.ebi.ac.uk/Tools/msa/muscle/), and the phylogenetic tree from these data was constructed by using the neighbor-joining method. The optimal tree with sum of branch length equal to 0.23440320 is shown. The percentage of replicate trees in which the associated taxa clustered together in the bootstrap test (1,000 replicates) are shown. The phylogenetic tree is drawn to scale; branch lengths in the same units as those of the evolutionary distances are used to infer the phylogenetic tree. The scale bar represents 0.02 substitutions per site. The evolutionary distances were computed by using the Kimura 2-parameter method and are presented as number of base substitutions per site. The analysis involved 16-nt sequences. All positions containing gaps and missing data were eliminated. The final dataset contained 71 positions. Evolutionary analyses were conducted by using MEGA6 (http://www.megasoftware.net). The accession numbers of each sequence are shown for the viruses. HeV, Hendra virus. NiV-B, Nipah virus Bangladesh strain; NiV-I, Nipah virus Indian strain; NiV-M, Nipah virus Malaysian strain.

To detect neutralizing antibodies against HeV and NiV, we used neutralization assays with infectious HeV and NiV (*14*) and pseudotyped vesicular stomatitis virus possessing NiV envelope proteins (*15*) (Table 2). Samples with positive results were subsequently tested by ELISA for IgM against NiV. Neutralizing antibodies against NiV and correspondingly lower neutralizing antibody titers against HeV were found for 3 patients. IgM against NiV was also detectable in these same 3 patients. The pattern of neutralizing antibodies and IgM in acute-phase and convalescent-phase serum samples is evidence of recent exposure to a henipavirus. A serum sample from 1 of these patients (obtained 5 days after clinical sign onset) was also positive by real-time PCR for NiV, and a single-sequence read (71 bp) of the P gene of NiV was detected from a MiSeq (http://systems.illumina.com/systems/miseq.html) next-generation sequencing run of a cerebrospinal fluid sample from another of these patients (Figure 2). This short segment had 99% nt identity with NiV isolates from Malaysia and 94%–96% identity with NiV isolates from Bangladesh. Further attempts to amplify additional genome and isolate the virus were unsuccessful. The short-read archive has been deposited in the DNA Data Bank of Japan (accession no. DRA002637). All serum samples from 4 dogs were positive for neutralizing antibodies against NiV. NiV neutralizing antibodies were not detected in samples from animals of any other species.

**Table 2:** Chronologic serologic test and nucleic acid detection results for 3 patients in NiV outbreak, Philippines, 2014*

Patient	Onset of clinical signs	Date of sample collection	IgM ELISA ratio	NiV SNT titer†	NiV SNT titer‡	Nucleic acid detection
1 (AES)	Apr 7	Apr 12	11.8	Neg	1:150	Pos (qPCR) from serum of Apr 12
		May 11	8.5	1:80	1:1,200	NA
		May 22	6.5	1:40	1:950	NA
2 (AES)	Apr 7	Apr 15	13.2 (6 am), 12.9 (3 pm)	1:10	1:200	Pos (NGS) from CSF of Apr 12
		May 8	11.3	1:80	1:2,600	NA
		May 21	9.1	1:20	1:1,800	NA
3 (ILI)	Apr 2	May 21	5	1:40	1:420	NA

*The cutoff for the IgM NiV ELISA is a ratio of 2, for SNT using infectious NiV is ≥1:4, and for pseudotype-based SNT is 1:80. All samples were serum except for the sample tested by NGS, which was CSF. AES, acute encephalitis syndrome; CSF, cerebrospinal fluid; ILI, influenza-like illness; NA, not applicable; Neg, negative; NGS, next-generation sequencing; NiV, Nipah virus; pos, positive; qPCR, quantitative PCR for NiV; SNT, serum neutralization test. †Test used infectious NiV. ‡Test used pseudotyped vesicular stomatitis virus.

## Conclusions

Clinical presentations, epidemiologic findings, and serologic results suggest that the virus causing this outbreak was a henipavirus. It was most likely NiV or a virus that is antigenically and genetically closely related to NiV.

Epidemiologic data suggest that the most common route of virus transmission to humans was direct exposure to infected horses, contact with contaminated body fluids during slaughtering of sick horses, and/or consumption of undercooked meat from infected horses. However, for at least 5 cases, clinical and epidemiologic evidence suggest direct human-to-human virus transmission. No protective equipment was used by those who cared for case-patients in the home, and health care workers used gloves and a face mask but not eye protection. The evidence of human-to-human transmission in this outbreak confirms the need for preventative measures in home care and health care settings.

Although the source of the horse infections is unclear, on the basis of the known ecology of henipaviruses, the most likely source is fruit bats (family *Pteropodidae*) (*10*). Bats belonging to this family were reported near at least 1 of the 2 villages.

Ongoing surveillance in the area and neighboring regions is needed to help with prompt response to future outbreaks. Activities should include accurate and rapid diagnosis of new outbreaks, investigation of risk factors associated with spillover and virus transmission, implementation of control measures, and further characterization of the virus involved.
